# Supplementary material for: Interferon-regulated genetic programs and JAK/STAT pathway activate the intronic promoter of the short ACE2 isoform in renal proximal tubules
Source: bioRxiv. 2021 Jan 19:2021.01.15.426908. Preprint. [Version 1] doi: 10.1101/2021.01.15.426908 (PMC7836111; doi:10.1101/2021.01.15.426908)

A

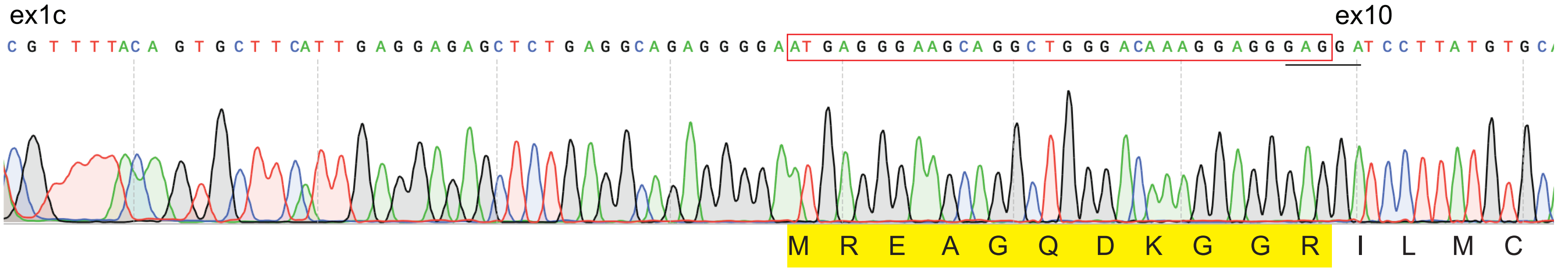

B

new promoter and exon1c

GACAAGGTATTTAACTTTCTTTGGTTTCAGTTTCCTTATTTTATAAAGTAGAATAGTAATTCCCAGGTTGCAGGCTTGTGAGA
GCCTTAGGTTGGATTCCCTAGCTTGAAAAGGAGATCGTTTTACAAGTGCTTCATTGAGGAGAGCTCTGAGGCAGAGGGGA
ATGAGGGAAGCAGGCTGGGACAAAGGAGGGAG

C

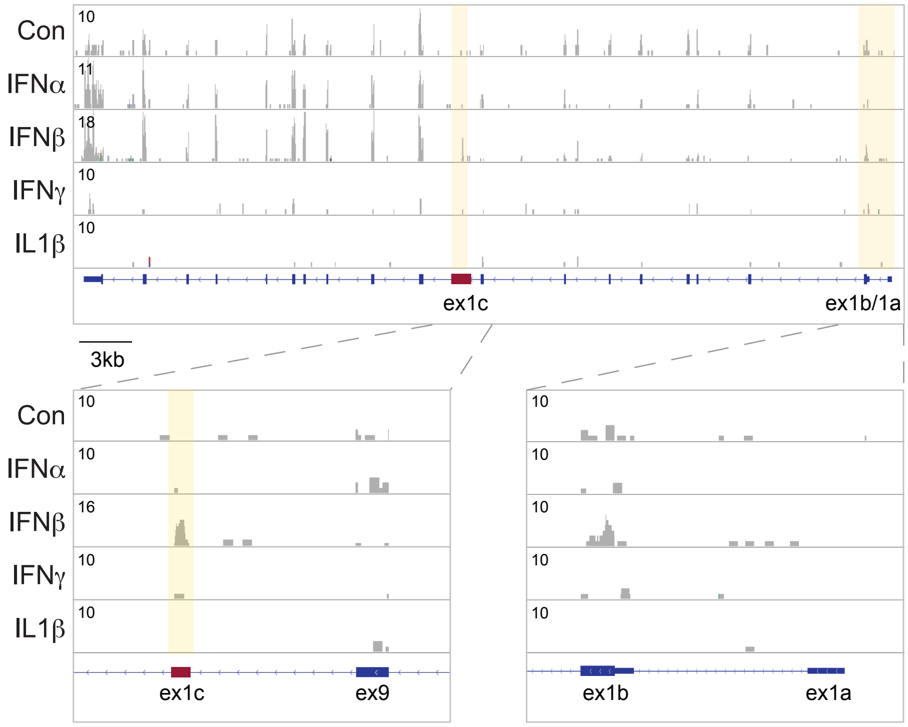

Supplement: Supplement 1 — Supplementary Figure 1. A novel short transcript of ACE2 was induced by interferons. (A) Sanger sequencing showed sequence at exon/exon boundaries of short ACE2 transcript exon1c-10. Amino acid translation is shown below. (B) The sequence of the first exon of dACE2. TATA boxes and TSS marked in blue and red, respectively, and CDS showed in the highlighted yellow. (C) IGV plot displayed RNA reads covering the entire ACE2 gene from control and experimental cells. [file media-1.pdf]
