# Supplementary figures and images for: Interferon-regulated genetic programs and JAK/STAT pathway activate the intronic promoter of the short ACE2 isoform in renal proximal tubules
Source: bioRxiv. 2021 Jan 19:2021.01.15.426908. Preprint. [Version 1] doi: 10.1101/2021.01.15.426908 (PMC7836111; doi:10.1101/2021.01.15.426908)

**A**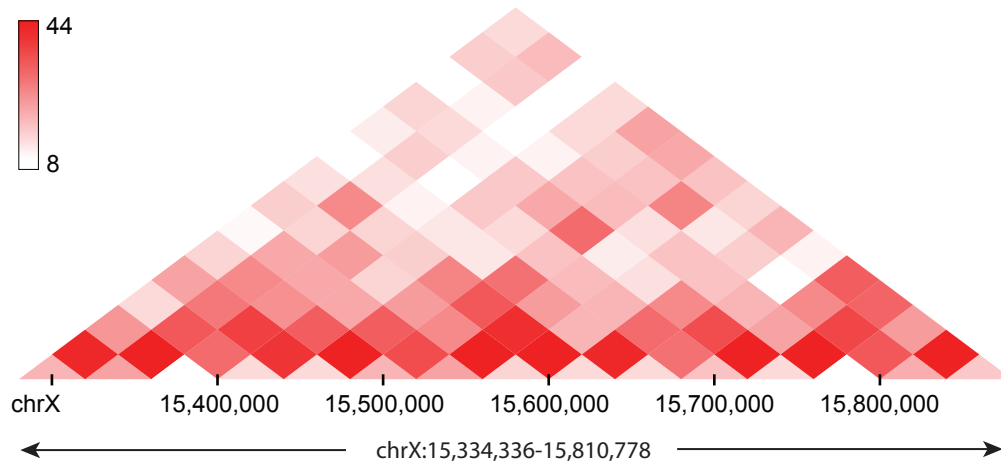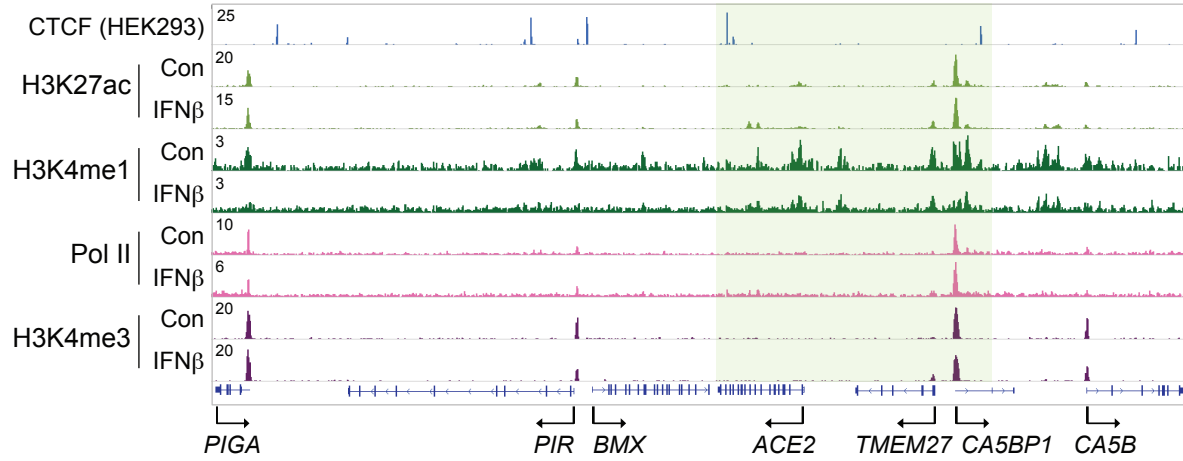

| RNA-seq     | <i>PIGA</i> | <i>PIR</i> | <i>BMX</i> | <i>ACE2</i> | <i>TMEM27</i> | <i>CA5BP1</i> | <i>CA5B</i> |
|-------------|-------------|------------|------------|-------------|---------------|---------------|-------------|
| Con         | 372.6       | 24.9       | 0.9        | 20.2        | 114.5         | 130.2         | 51.0        |
| IFN $\beta$ | 388.9       | 21.6       | 2.3        | 155.6       | 499.0         | 105.9         | 57.8        |
| Induction   | 1.0         | 0.9        | 2.6        | 7.7         | 4.4           | 0.8           | 1.1         |

Supplement: Supplement 2 — Supplementary Figure 2. Sub-TADs defined by Hi-C from human adrenal gland and ChIP-seq data from HEK293 cells in the 470kb locus including ACE2 and neighboring genes. mRNA levels of genes in the locus under the control of IFNβ measured by RNA-seq. [file media-2.pdf]

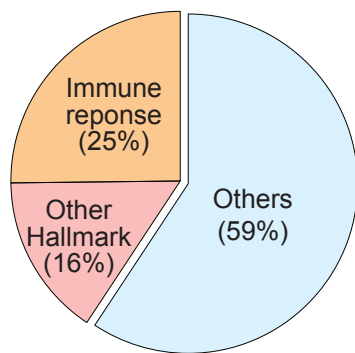

Supplement: Supplement 3 — Supplementary Figure 3. Percentage of induced gene classes via Hallmark Gene Sets (FDR q-value < 0.05). [file media-3.pdf]
